# Multiphoton intravital microscopy in small animals: motion artefact challenges and technical solutions

**DOI:** 10.1111/jmi.12880

**Published:** 2020-03-05

**Authors:** D. SOULET, J. LAMONTAGNE‐PROULX, B. AUBÉ, D. DAVALOS

**Affiliations:** ^1^ Centre de recherche du CHUL Department of Neurosciences Quebec Canada; ^2^ Faculty of Pharmacy Université Laval Quebec Canada; ^3^ Department of Neurosciences Lerner Research Institute Cleveland Clinic Cleveland Ohio U.S.A.

**Keywords:** Active compensation, animal restraint, gated acquisition, image processing, image registration, living animal, motion artefact, software, tissue immobilization, troubleshooting

## Abstract

Since its invention 29 years ago, two‐photon laser‐scanning microscopy has evolved from a promising imaging technique, to an established widely available imaging modality used throughout the biomedical research community. The establishment of two‐photon microscopy as the preferred method for imaging fluorescently labelled cells and structures in living animals can be attributed to the biophysical mechanism by which the generation of fluorescence is accomplished. The use of powerful lasers capable of delivering infrared light pulses within femtosecond intervals, facilitates the nonlinear excitation of fluorescent molecules only at the focal plane and determines by objective lens position. This offers numerous benefits for studies of biological samples at high spatial and temporal resolutions with limited photo‐damage and superior tissue penetration. Indeed, these attributes have established two‐photon microscopy as the ideal method for live‐animal imaging in several areas of biology and have led to a whole new field of study dedicated to imaging biological phenomena in intact tissues and living organisms. However, despite its appealing features, two‐photon intravital microscopy is inherently limited by tissue motion from heartbeat, respiratory cycles, peristalsis, muscle/vascular tone and physiological functions that change tissue geometry. Because these movements impede temporal and spatial resolution, they must be properly addressed to harness the full potential of two‐photon intravital microscopy and enable accurate data analysis and interpretation. In addition, the sources and features of these motion artefacts are varied, sometimes unpredictable and unique to specific organs and multiple complex strategies have previously been devised to address them. This review will discuss these motion artefacts requirement and technical solutions for their correction and after intravital two‐photon microscopy.

## Introduction

Two‐photon laser‐scanning microscopy was developed in 1990 and has become the method of choice to investigate biological processes in live animals, notably because its nonlinear nature confers it several appealing features over one‐photon confocal microscopy (Denk *et al*., 1990). Indeed, for the excitation process to occur at the sample two photons must be coincident both in space and time on the fluorescent molecule on an extremely short time scale. Given the low probability of such an event occurring, the photon density arriving at the sample must be enormous. This is possible with mode‐locked lasers providing a train of pulses of short duration (∼100fs) with high peak power and repetition rate (∼80 MHz) of near‐infrared light. Notably, the latter provides better tissue penetration than its visible counterpart and makes it especially suitable for thick tissue imaging. Moreover, since two photons are required to produce signal the latter scales with the square of laser power, hence no fluorescence is generated outside the focal volume and unwanted background is minimized. In addition, the detection layout in two‐photon microscopy does not require a physical pinhole since no fluorescence is generated outside the focus in the first place (Sanderson *et al*., 2014). This enables scattered photons to contribute to the useful signal since they originate from the focus by virtue of the nonlinear process described above. Therefore, two‐photon microscopy is highly appealing for intravital studies and possesses a remarkable ability to access moderately unperturbed environments with excellent temporal resolution facilitating the observation of dynamic biological events over long periods of time (Bullen, 2008; Niesner & Hauser, 2011; Pittet & Weissleder, 2011; Ellenbroek & van Rheenen, 2014). However, the extent to which the environment is perturbed is highly dependent on the location of the organ to be imaged. Indeed, gaining optical access deep into tissues that are themselves isolated creates substantial challenges in acquiring high‐quality images in a minimally invasive manner (Helmchen & Denk, 2005; Grewe & Helmchen, 2009; Megens *et al*., 2011). For example, the liver, kidney, gut and spleen are relatively simple to image because they can be readily exteriorized and placed under an objective lens. By contrast, the brain, spinal cord, heart and lungs are much more difficult to image, because they require elaborate surgical procedures that likely impact their functions. These challenges can create a thin line between successfully imaging a physiological process in a remote organ and observing unwanted direct or indirect effects of an intrusive procedure on an organ. Therefore, to conduct intravital imaging experiments, we must find a balance between minimizing invasiveness during sample preparation and preserving our ability to reliably record physiological phenomena at high resolution. The latter is especially challenging in living animals, because – regardless of their location in the body – all organs are inherently subjected to disturbances caused by various physiological processes, such as the heartbeat, respiratory cycles, vascular tone or peristalsis. These disturbances inevitably cause motion artefacts that corrupt frames and must be eliminated or compensated to properly and accurately analyze data (Lucotte & Balaban, 2014; Vinegoni *et al*., 2014). Researchers have recently begun devoting considerable effort into limiting the burden of motion artefacts. In this review, we will summarize technical and software solutions, particularly those that apply to studies in neuroscience, immunology and cell biology, and provide an overview of the organ‐specific tools available for stabilizing intravital optical imaging in small animals (mostly in mice, but also in rats and rabbits).

### The burden of motion artefacts

For intravital imaging at the cellular level, we must take into account certain challenges, such as physical access to the organ, tissue penetration and motion artefacts not typically encountered *in vitro*. Motion artefacts are arguably the most cumbersome to tackle, mostly because each organ has a unique pattern, frequency or amplitude of movement and an unpredictable nature. In addition, because tissue movement is not bound to rigid, planar translations, the experimenter may not notice physical deformations, such as rotation and scale changes, until the end of the imaging session, which can further complicate their analysis. Moreover, motion artefacts are prone to providing erroneous impressions of movement within the organ that are not necessarily caused by a biological process, but, rather, are false positives that must be considered during data analysis and interpretation. This phenomenon is amplified when small motile structures, such as microglial processes, growing axons or dendritic spines, are observed at high magnification, when minor disturbances in the imaging (XY) or axial (Z) planes can mislead the representation of their genuine displacements. This effect is crucial for morphological, speed or any dynamic analysis of phenomena in moving environments. Several strategies address these issues and will be discussed in the following sections, including their potential use, combination and applicability to different organs and types of artefacts. We will also discuss compensation approaches, such as organ immobilization, active compensation, gated/triggered acquisition and image processing.

## Animal restraint and tissue immobilization

In general, the extent of motion artefacts is heavily influenced by the immobilization strategy used to restrict displacement of the animal and organ of interest while avoiding perturbations to its normal functions. The ideal scenario involves minimally invasive surgery to access the sample and preserve its physiological environment. Because the location of an organ dictates the best approach to minimize shifting, the following three sections discuss many scenarios for physical restraint of abdominal organs (kidney, spleen, liver and intestine), organs located inside/close to the chest cavity (heart, lung and spinal cord) and the brain, which is uniquely located within the skull, away from extensive movements caused by breathing and heartbeat. Typical examples of animal restraint strategies are described in Table 1.

**Table 1:** Available restraining approaches for TP‐IVM

Organ	Biological events observed	Restraining method	Refs
Gut	‐ Immune cell trafficking ‐ Myenteric neurogenesis ‐ Mucosal homeostasis and cellular interactions	‐ Externalized gut, pinned, sutured or sandwiched ‐ Gut stabilized with animal's weight ‐ Topical application of papaverine	(Klinger *et al*., 2012; Xu *et al*., 2012; Goto *et al*., 2013; Mizuno *et al*., 2014; Motegi *et al*., 2020)
Abdominal organs	‐ Gene expression ‐ Cellular trafficking	‐ Exteriorized organ ‐ Custom microstage device ‐ Abdominal imaging window	(Denk *et al*., 1990, Ritsma *et al*., 2014)
Spinal cord	‐ Resident and peripheral immune cell behaviour trafficking ‐ Extravasation and interactions with tissue ‐ Vascular permeability ‐ Axonal growth	‐ Spinal cord clamps ‐ Implanted custom Chamber ‐ Embedding with agar	(Kerschensteiner *et al*., 2005; Odoardi *et al*., 2007; Johannssen & Helmchen, 2010; Kim *et al*., 2010; Nikić *et al*., 2011; Davalos & Akassoglou, 2012; Farrar *et al*., 2012; Fenrich *et al*., 2013; Coisne *et al*., 2013; Evans *et al*., 2014; Schaffran *et al*., 2019), (Figley *et al*., 2013)
Lung	‐ Immune cell interactions ‐ Leukocyte trafficking and extravasation	‐ Cover slip over exposed lung ‐ Thoracic suction window ‐ Endotracheal tube	(Kreisel *et al*., 2010; Fiole *et al*., 2014; Veres *et al*., 2017), (Looney *et al*., 2011, Ueki *et al*., 2018)
Heart	‐ Leukocyte recruitment and trafficking dynamics ‐ Cell‐endothelium interactions ‐ Collagen‐rich structures	‐ Ring‐shaped stabilizer ‐ Compressive cover slip ‐ Gluing device	(Li *et al*., 2012; Lee *et al*., 2012; Diaz *et al*., 2014; Matsuura *et al*., 2018)
Brain	‐ Neuronal plasticity ‐ Ca^2+^ imaging ‐ Neuronal activity ‐ Vascular structure and permeability ‐ Electrophysiological recordings ‐ Axonal growth ‐ Glial cell behaviour	‐ Stereotactic frame ‐ Head plate ‐ Heat post clamps ‐ Ear‐fixed bars ‐ Skull glued to support	(Zhang *et al*., 2012; Manglani & McGavern, 2018), (Dorand *et al*., 2014, Alieva *et al*., 2014), (Drew *et al*., 2010; Shih *et al*., 2012), (Mizrahi *et al*., 2004, Gu *et al*., 2014)

### Gut and other abdominal organs

Physical stabilization of abdominal organs is much easier when they are exteriorized (Marques *et al*., 2015), even though this approach is considerably invasive. These organs can be positioned in almost any configuration, which enables whole‐organ imaging while avoiding the need to observe limited sections. However, this approach comes at the expense of larger degrees of freedom for movement and, thus, requires appropriate stabilization devices. Conventionally, organs are placed in a custom holder and pressure is applied with a cover slip or they can be sandwiched between two cover slips so that imaging is performed onto a planar surface (Klinger *et al*., 2012; Xu *et al*., 2012; Goto *et al*., 2013; Mizuno *et al*., 2014; Motegi *et al*., 2020). Although this approach limits large‐scale XY or Z drift, it is unlikely to be suitable for smaller shifts. Recently, researchers combined this setup with single‐photon confocal microscopy for intravital imaging of the mouse liver, a relatively low‐cost alternative to two‐photon microscope systems (Soulet *et al*., 2013). This approach can be used for intravital microscopy depending on the tissue. However, in the mouse liver, depth imaging is limited to approximately 100 μm from the organ surface (Marques *et al*., 2015). Deeper imaging into living organs, requires limiting the extensive light scattering and absorption that are inherent with single photon excitation, but limited when two‐photon excitation is used (Helmchen & Denk, 2005; Cao *et al*., 2012). Cao and colleagues developed a microstage device that uses ball joints to freely translate and rotate partially externalized organs in three dimensions (3D) (Megens *et al*., 2010). When exteriorizing abdominal organs, one should consider several practical issues, including carefully regulating the chamber's temperature and providing physiological fluids that prevent organs from drying out. The use of an inverted microscope can provide additional benefits, as the animal's weight can be used to apply an even force on the organ to help dampen motion (Xu *et al*., 2012; Mizuno *et al*., 2014).

Despite reducing overall movements, physical immobilization of exteriorized organs will likely not compensate for stable high‐resolution imaging (Soulet *et al*., 2013). To increase stability, glue or sutures are often used, although care must be taken to prevent unwanted immune activation and perturbations to the environment (Goto *et al*., 2013; Mizuno *et al*., 2014). Even then, triggered acquisition or image processing might be required. For example, in the gut, peristalsis causes unpredictable, high‐amplitude movements that are not fully compensated by physical restraint alone (Chèvre *et al*., 2014; Ritsma *et al*., 2014). Goto *et al*. used papaverine, an opium alkaloid that treats spasms of the gastrointestinal tract, to suppress movement of the ileum (Goto *et al*., 2013), which might affect intestinal functions in unpredictable ways.

Alternatively, implanted chambers can adequately stabilize organs and allow imaging of the same region for extended periods of time. For example, Ritsma and colleagues described an implant that provides optical access to the kidney, liver, intestine, pancreas or spleen for up to 5 weeks (Davalos *et al*., 2008). By securing organs to the inner wall of the chamber with glue and using a custom‐made imaging box, they reduced movements during data acquisition in a modestly invasive fashion. Moreover, placement of sterile gauze between organs and rigid structures, such as the diaphragm or rib cage, can help decrease breathing artefacts. However, since inflammatory reactions are difficult to avoid outside the chamber, appropriate controls are required to ensure that the implant does not change the organ's functions.

### Organs located inside/close to the chest cavity

#### Spinal cord

Organs located inside (heart, lungs) or close to (spinal cord) the chest cavity are the most challenging to tackle in terms of movement compensation. However, the spinal cord is notably unique because it is physically confined in the vertebral column and thus benefits from rigid support that helps reduce macroscopic movements. Because breathing generates most movements in the spinal cord, every effort should be made to promote smooth respiration, including careful choice of anesthetics and animal positioning (Kerschensteiner *et al*., 2005; Odoardi *et al*., 2007; Johannssen & Helmchen, 2010; Davalos & Akassoglou, 2012). As physical *restraint* of the spinal cord itself is not possible, vertebrae can be targeted to dampen its movement. For example, some studies described a stabilization method for short‐term imaging in which mice are suspended with spinal clamps to elevate the abdomen and allow them to breath freely (Johannssen & Helmchen, 2010; Nikić *et al*., 2011; Coisne *et al*., 2013; Schaffran *et al*., 2019). Since residual motion artefacts caused by heartbeat and breathing are generally not eliminated altogether, they can be further reduced by embedding the spinal cord in agar (Kerschensteiner *et al*., 2005). This strategy is widely used to image large regions of interest for short periods of time and can be applied to many segment of the spinal cord (Davalos *et al*., 2012; Aubé *et al*., 2014; Evans *et al*., 2014; Haghayegh Jahromi *et al*., 2017). Selecting different regions of the spinal cord for imaging also poses different challenges and advantages. For example, the thoracic spinal cord naturally curves outwards from the animal's body and thus requires less muscle displacement for exposure; but it is also closer to the heart and lungs, making it more sensitive to movement artefact. In the lumbar spinal region, the larger separation between the cord and surrounding vertebrae reduces the risk of puncturing or damaging the cord during laminectomy. However, it is covered by a thicker muscular layer due to its inward curvature, thus requiring a more invasive surgical procedure.

Indeed, to obtain optical access to the specimen for imaging, one must expose the area to be imaged by removing or preferably displacing tissue and/or bone covering the spinal cord. This needs to be repeated for longitudinal imaging experiments of the same cells or spinal cord areas, for every imaging session (Johannssen & Helmchen, 2010). Several approaches have been devised and used to carry out repetitive imaging of the mouse spinal cord. Experiments that require only one or two imaging sessions can be performed by imaging through the interlaminar space, particularly in the thoracic spinal cord, provided of course that the limited field of view is compatible with the experimental design (Kim *et al*., 2010; Farrar *et al*., 2012). For longer‐term imaging requiring several reimaging sessions, the best strategy is to use a chronic implant to reduce the risks associated with repetitive surgical interventions. For example, Farrar *et al*. robustly stabilized animals with V‐shaped stainless‐steel clamps that sandwich vertebrae (Nikić *et al*., 2011; Fenrich *et al*., 2013). This design is limited by the relative complexity of construction, modest success rate and the need to administer antiinflammatory or immunosuppressant drugs to animals to prevent fibrosis over the laminectomy site. Alternatively, Fenrich *et al*. developed a window‐chamber design with staples and paperclips as supporting elements. This approach yields moderately higher success rates, longer imaging periods and is easier to use than the design by Farrar *et al*. (Figley *et al*., 2013). Finally, Figley *et al*. designed a transparent 3D‐printed window built from nonmetallic materials for compatibility with nonfluorescent or laser‐scanning imaging modalities such as optical coherence tomography and photoacoustic imaging (Veres *et al*., 2017a). These window designs support stable intravital imaging of the mouse spinal cord; however, they can hardly eliminate minute drifts of the spinal cord and microscopic displacements caused by vascular tone, which can be corrected with image processing.

#### Heart and lungs

The heart and lungs present unique challenges for physical restraint. Because their complete immobilization is not possible, restraining methods aim to reduce movements and thereby facilitate the processing of image sequences offline. In addition, with gated acquisition, cardiac rhythm and breathing activity can be monitored to help synchronize image collection with periods of minimal organ movement. This approach will be discussed further in later sections.

To access the heart and lungs, the chest cavity must be opened, which introduces challenges beyond surgical intervention. Inside the thorax, there is a vacuum created by the moving diaphragm to force air flow in and out of the lungs. Disrupting this vacuum to access the heart or lungs without respiratory support will eventually cause the lungs to collapse. Hence, to access those organs, one must consider the unique environment inside the thorax and maintain the vacuum with appropriate sealing and mechanical ventilation. A first approach was to use an endotracheal tube to stabilize the trachea during imaging (Fiole *et al*., 2014). However, this method is not ideal to maintain the physiological integrity of the trachea wall and mucosa. New methods have therefore been developed to optimize the imaging window directly in the opening of the thoracotomy with a trachea holding device and a glued glass cover slip (Kreisel *et al*., 2010; Looney *et al*., 2011; Veres *et al*., 2017b). To collect image frames with minimal artefacts, this approach must be combined with data acquisition synchronized with respiration. As a more invasive, yet robust, alternative, a thoracic window can minimize movements and stabilize imaging opportunities. This type of window was introduced in 1939 for imaging of the cat lung and was revisited for the mouse in 2011 by Looney and colleagues based on Wagner's design for the dog (Ueki *et al*., 2018). The suction‐based device applies gentle pressure (20–30 mm Hg) onto the lung so that it stays loosely adhered to the organ and allows it to expand and retract freely (Lee *et al*., 2012). Residual movements in 3D are substantially reduced to a magnitude of 5–10 μm, which enables high‐resolution imaging of immune trafficking and dynamics. This thoracic window permits imaging during the entire respiratory cycle without interrupting the ventilation supplied to the animal, which is highly desirable, especially during prolonged imaging sessions. The caveats related to this approach include invasiveness, technical difficulty and the fact that mice are not spontaneously breathing.

Similarly, the beating heart has high‐magnitude rhythmic movements that create considerable challenges for stable high‐resolution imaging. Most methods that stabilize the heart share the same principle: lightly gluing the organ to a rigid support and monitoring the animal's electrocardiogram and/or respiratory function and trigger data acquisition during periods with less movement. This period generally occurs during diastole, when the heart temporarily relaxes before the next systole. For example, Lee and colleagues developed a ring‐shaped stabilizer whose base is fixed to the myocardium, which significantly dampens large‐amplitude movements; however, cardiac contractions pressing against the device create residual motion artefacts (Aguirre *et al*., 2014; Matsuura *et al*., 2018). Jones *et al*. imaged single cardiomyocytes with a similar setup to that of Aguirre *et al*. These authors also administered the neuromuscular blocker pancuronium to mice and rabbits to reduce breathing‐induced movements (Li *et al*., 2012; Diaz *et al*., 2014; Jones *et al*., 2018). However, care must be taken when using muscle‐paralyzing agents because they can induce considerable changes in heart rate or blood pressure (Li *et al*., 2012). Li *et al*. successfully imaged neutrophil trafficking in beating transplanted hearts by securing the mouse between two metal plates and pressing on the organ with a cover slip (Drechsler *et al*., 2010). The major arteries surrounding the heart are also of interest in many pathological conditions, especially atherosclerosis (Chèvre *et al*., 2014; Ritsma *et al*., 2014; Manglani & McGavern, 2018). Chèvre *et al*. performed multichannel, high‐speed imaging of the isolated carotid artery, using a bevel support and by pressing against the vessel with a cover slip, to collect high‐resolution images of atherosclerotic lesions in mice (Ritsma *et al*., 2014). Exposed carotid arteries were also observed without a specific restraining apparatus; however, to obtain stable images, fast, gated acquisition had to be performed, in conjunction with extensive processing and at the expense of spatial resolution (Chèvre *et al*., 2014; Manglani & McGavern, 2018). These techniques can be combined with heart rate‐ and breathing‐triggered acquisition, which will be discussed in more detail in a later section.

#### Brain

Similar to the spinal cord, the brain is tightly contained by a bone structure that naturally provides physical restraint. Its remoteness from the heart and chest cavity also helps create an environment moderately affected by motion artefacts. Physical restraint of the brain itself is futile, because it must be allowed to move freely in the subarachnoid space; therefore, techniques that stabilize the skull have the sole purpose of stably supporting the organ for imaging. Two approaches have been developed towards this end: open‐skull and thinned‐skull windows. As their names imply, the first involves a complete craniotomy, whereas the second does not. Since the brain was the first organ imaged *in vivo* by two‐photon microscopy over 20 years ago, both methods have been used extensively by researchers in the neuroscience community and implementation has been extensively described (Xu *et al*., 2007; Zhang *et al*., 2012; Alieva *et al*., 2014; Dorand *et al*., 2014). Each method has its own merits and drawbacks, and selecting one over the other depends on experimental design and goals; for example, thinned‐skull windows allow imaging of the intact mouse cortex over several hours, but can cause pial injury if they are rethinned more than two to three times. Conversely, open‐skull windows allow for numerous reimaging sessions over days to weeks or months, but their original implantation causes substantial pial inflammation that requires at least 10 days to subside and allow for ‘physiological’ imaging conditions (Shih *et al*., 2012). From an image quality perspective, the skull thinning procedure can cause irregularities that induce spherical aberrations and are thus more challenging to perform successfully than open‐skull windows. Recent attempts have been made to combine the best qualities of both methods by creating a thinned‐skull window that is polished and reinforced by a glass cover slip that is glued over the thinned area to prevent bone regrowth (Mizrahi *et al*., 2004; Drew *et al*., 2010; Dorand *et al*., 2014). Of interest is that brain imaging methods have also been developed for *in vivo* study of deeper brain structures such as the hippocampus in mice, as this region is of extensive interest for both physiological and pathological brain functions (Gu *et al*., 2014). This method is also quite challenging, since it requires surgical removal of the cortical tissue above these deeper brain regions, and even longer waiting periods than those for window implantation for inflammation resolution (Park *et al*., 2019). All these methods stabilize the organ, using a common fixation approach through either a stereotactic frame or a head‐restraining apparatus. Moreover, translucent substances, such as silicone elastomer or low‐melting point agarose, can fill the space between the cortical surface and cover slip to help maintain their adhesion over extended periods of time (Holtmaat *et al*., 2009; Yildirim *et al*., 2019). These substances also minimize the refractive‐indices mismatch between the cover glass and brain tissue, thereby reducing optical aberrations. Generally, brain‐related motion artefacts do not create a major issue in anesthetized mice because of their low amplitude and frequency, ranging from about 2 to 5 cycles/s. However, insufficient anesthesia induces accelerated breathing and heartbeat that translate into visible drift and artefacts. We stress that the level of anesthesia must be carefully monitored during the entire experiment (i.e. surgery and imaging) to avoid pain and distress to animals.

Intravital imaging of the brain becomes a bigger challenge in awake behaving animals, in which tissue displacements are more pronounced and difficult to tackle because their amplitude is likely larger than the structures under observation. However, observation of the brain of nonanesthetized animals with optical microscopy better associates behaviours or external stimuli to communication patterns among neuronal populations. For this purpose, calcium (Ca^2+^) transients and action potentials are generally recorded with genetically encoded probes, adeno‐associated virus vectors or by bulk loading fluorescent indicators into the brain (Garaschuk *et al*., 2006; Andermann *et al*., 2011; St‐Pierre *et al*., 2014).

The volume spanned by neuronal networks in specific brain compartments is substantial. Thus, the ideal experimental paradigm enables fast, stable, sensitive and high‐resolution imaging of neuronal structures over large areas and depths. However, for practical and technical considerations these features are hard to achieve simultaneously *in vivo*, so precedence must be given to some over others. The ideal paradigm primarily discriminates the approaches developed to capture neuronal activity in awake animals, which can be loosely sorted into tabletop and portable systems. Tabletop systems usually involve head‐fixed mice under a conventional microscope and a laser source that allows two‐photon excitation and its associated benefits. In these mice, access to the brain and its restraint are similar to anesthetized mice; however, awake animals are exposed to different environments suitable for studying the motor, visual, auditory or barrel cortices, such as during locomotion or sensory stimulation (Sato *et al*., 2007; Kerlin *et al*., 2010; Scott *et al*., 2014; Nadella *et al*., 2016).

Recent technical developments use new microscope technology to decrease motion artefact by increasing the speed of acquisition. For example, random‐access scanning microscopy enable high‐speed volumetric imaging of brain with reduces intraframe motion artefacts (Botcherby *et al*., 2006). Another available technic is the use of a Bessel beam to reduce the acquisition time in the optical Z‐axis by increasing the length of the point spread function of the focused excitation beam to generate an extended depth of field image (Song *et al*., 2017). Recently, Song *et al*. created a method to address loss of depth information limitation of Bessel beam by splitting the beam. Referred as volumetric Two‐photon Imaging of Neurons using Stereoscopy, the two excitation beams create a stereoscopic ‘V’‐shaped point spread function configuration that preserves depth information. The combination of Bessel beam and volumetric Two‐photon Imaging of Neurons using Stereoscopy allows a high‐speed image acquisition and a lower exposure time without loss of 3D information (Birkner *et al*., 2017). Another method using ultra‐short laser pulses can increase rapidly the fluorescent indicator energy with lower power laser. This method presents another solution to increase the acquisition speed, to decrease the phototoxicity and to increase the depth penetration (Sato *et al*., 2017).

Recently, researchers performed dual‐axis Ca2+ imaging in distinct areas of the visual cortex using gradient refractive index microendoscopes and optogenetic perturbation of targeted neuronal networks. This was performed alongside Ca^2+^ imaging that probed the activity in each area during different activity states (Lecoq *et al*., 2014; Packer *et al*., 2015). Interestingly, along with image registration, Packer and colleagues used microscope parts that moved the optical path relative to the sample to reduce the impact of motion artefacts (Gulati *et al*., 2017). This paradigm is also common in portable‐microscope systems that fix fibre‐based and integrated microscopes to the animal's head, thereby allowing for behavioural testing of the mice during imaging, since they are not head‐restrained and can move freely (Ghosh *et al*., 2011; Helmchen *et al*., 2013; Ziv *et al*., 2013; Chen *et al*., 2018). Moreover, integrated microscopes with light‐emitting diode (LED) illumination and a large depth of field, enable experimenters to perform video‐rate image acquisition of extensive fields of view with fewer effects from motion artefacts (Ziv *et al*., 2013). When equipped with a microendoscope, miniaturized microscopes support relatively stable Ca^2+^ imaging in deep brain areas, such as the hippocampus, while animals are awake and behaving (Kerr & Nimmerjahn, 2012; Helmchen *et al*., 2013; Berdyyeva *et al*., 2014). Without restraining devices or anesthetic, miniature microscope avoids movement of the head and cause less intraframe motion artefacts (Sawinski *et al*., 2009; Zong *et al*., 2017). The main drawbacks associated with these systems are their limited spatial resolution and depth of field, as well as a limited single‐photon/colour excitation and the impossibility of moving the lens during imaging. These drawbacks will undoubtedly be addressed in the near future and should render integrated microscopes as widespread, complementary tools to two‐photon excitation systems mainly for the study of the brain and the spinal cord (Paukert & Bergles, 2012; Sekiguchi *et al*., 2016; Nelson *et al*., 2019).

## Gated acquisition and active compensation

Despite physical restraint, motion artefacts will likely hinder high‐resolution imaging in organs prone to high‐amplitude displacements. Gated acquisition and active compensation can help address this and provide stable‐imaging conditions, albeit with reduced temporal or spatial resolution. Representative examples are provided in Table 2.

**Table 2:** Gated acquisition and active compensation systems

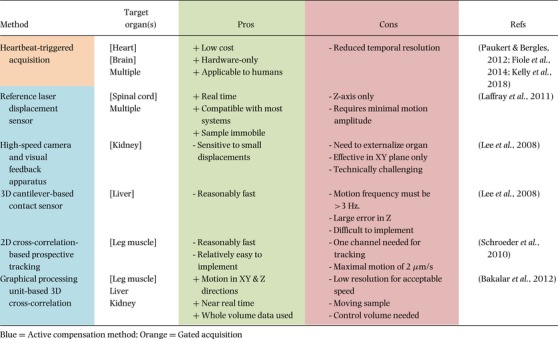

### Gated acquisition

Gated acquisition collects images at dedicated time points, when movement amplitude or frequency is minimal. This method is generally used to monitor the heart and lungs, because it can precisely monitor their activity and perform imaging during specific cycles of heartbeat, respiration or both. This idea supports *prospective* gating. Although gated acquisition helps acquire images without major artefacts, its temporal resolution is compromised and, thus, can overlook phenomena that occur outside of these ‘stable’ cycles. Nevertheless, this approach is relatively simple to implement and can address many biological questions. Interestingly, Paukert and Bergles showed that microdisplacements occurring in the brain were primarily caused by heartbeat. Based on this finding, they used electrocardiogram‐triggered interlaced scanning to observe dendritic spines (Jenkins *et al*., 2013).

An alternative method to prospective gating involves acquiring as many images as possible while monitoring the animal's electrocardiogram or breathing activity and then selecting frames that were recorded during the cycles of interest. The throughput is even higher when the microscope is equipped with a fast‐scanning system, such as a resonant scanner or spinning polygonal mirror. This approach, coined *retrospective* gating, revealed high‐resolution interactions between macrophages and dendritic cells in the mouse lung without stabilizing the organ. Of note is that only one image was selected every minutes within 5 s‐acquisition periods, which decreased temporal resolution, but created stable image sequences with good spatial resolution (Kreisel *et al*., 2010). As a complement to gated acquisition, the heart can be paced with electrodes or infrared laser light to dictate heartbeat frequency as shown here in rabbits and mouse (Laffray *et al*., 2011; Kelly *et al*., 2018). However, these procedures require special equipment and change the normal function of the heart, which would not be suitable for many intravital imaging purposes.

### Active compensation

Motion artefacts that stem from relatively high‐amplitude movements can be detected and corrected in real‐time with a number of hardware and software solutions. This concept supports active compensation, in which the microscope stage and/or objective lens move to accommodate sample displacements in 3D. Because microscope stages have high inertia, moving the objective lens is inherently faster and allows the sample to remain stationary, which can be critical when sudden movements occur. A contact‐free sensor of optical displacement has been described to actively synchronize the objective's axial position with the moving surface of the rat spinal cord, enabling stable Ca^2+^ imaging *in vivo* (Lee *et al*., 2008a). With this approach, the circular shape of the reference laser beam becomes hemicircular as it moves away from focus, thus providing an optical means to monitor the position of the tissue with respect to that of the objective. In this case, solely adjusting the objective's axial position (perpendicular to the imaging plane) implies more steps to compensate for X‐Y displacements. One group used a piezoactuator‐driven pentagon with a high‐speed loop that provides visual feedback by tracking beads in the kidney and compensating for planar displacements (Lee *et al*., 2008b). This group also added a cantilever‐based sensor to their system to guide objective displacement in the axial direction (Schroeder *et al*., 2010). Despite notably reduced amplitude of X‐Y movement, this approach requires contact with the sample, which lessens compensation for vertical motion and relies on charge‐coupled device camera detection. Therefore, this approach would require extensive hardware modifications to be applied to two‐photon excitation microscopy.

Methods based on image analysis can also actively monitor changes in reference volumes and adjust the position of the stage and/or the objective lens in real time (Bakalar *et al*., 2012; Lee *et al*., 2014). Schroeder and colleagues’ design is based on 2D cross‐correlation between two orthogonal imaging planes in a given volume and can correct for X‐Y displacements only with a speed limited to 2 µm/s ^(^Bakalar *et al*., 2012). The authors improved the performance of their system with resonant scanners for video‐rate acquisition coupled to real‐time calculations of 3D cross‐correlation between volumes of interest over time (Lee *et al*., 2014). They performed calculations on graphical processing units to estimate tissue motion from entire z‐stacks (i.e. in 3D), providing considerably more information for the motion compensation algorithm than 2D‐imaging planes. However, this gain in accuracy comes at the expense of increased computational time, implying that high‐resolution images might need to be scaled‐down to keep up with the large amount of data generated by the resonant scanner. This issue will undoubtedly be addressed in the near future. This innovative approach is also limited by the displacement range of the objective (limited to about 150 µm) and less‐efficient motion compensation for displacement rates greater than 200 µm/min, which might preclude its use in some organs, such as the gut or heart (Lee *et al*., 2014).

## Image processing

Postprocessing is generally an integral part of intravital imaging experiments. It is indispensable for most organs despite best efforts to suppress and compensate for tissue motion; however, the amount of processing required varies by organ and usually depends on the structures observed and type of data to be harvested. Some software solutions with key features for two‐photon intravital microscopy are suggested in Table 3.

**Table 3:** Available software and algorithms for TP‐IVM image processing

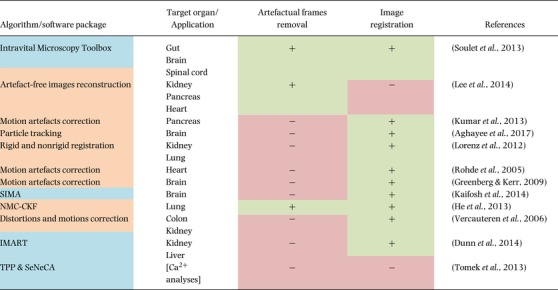

Orange = algorithms requiring programming skills; Blue = Graphic User Interface available; Green = implemented feature; Red = Not implemented feature.

Abbreviations: IMART, intravital microscopy artefact reduction tool; NMC‐CKF, nonlinear motion compensation algorithm using a cubature Kalman filter; SeNeCA, Search for Neural Cells Accelerated; SIMA, Sequential IMaging Analysis.

Commonly used operations include image filtering for noise reduction, artefact removal, features detection and image reconstruction and registration (alignment). Different experimental paradigms call for many image‐processing approaches. Of note is that image reconstruction and registration do not provide universal solutions to compensate for any type of artefact. Instead, the organs under observation, as well as the amplitude and time scale over which artefacts occur, dictate the optimal method to use. When movement amplitude is large and precludes regions of interest that will be registered between successive frames, whole‐frame removal is generally required, as often occurs when imaging the gut, heart, lung and spinal cord. Furthermore, different similarity metrics can be applied to multidimensional image sequences to automatically remove unwanted frames according to user‐defined parameters after acquisition is complete (Soulet *et al*., 2013). Alternatively, piecewise image reconstruction can be performed offline to stitch together artefact‐free image segments from different times during acquisition (Santamaría‐Pang *et al*., 2015). This approach reduces temporal resolution, but it also allows image collection in moving organs without the need for triggered acquisition schemes and complex instrumentation. As another option, the machine learning revolution makes it possible to create algorithms for image reconstruction predicting morphological structures. Even if it needs a specific algorithm for a specific structure recognition, the possibility of segmenting the object of interest within the surrounding noise allows a high accuracy for 3D reconstruction (Kumar *et al*., 2013; Haft‐Javaherian *et al*., 2019).

Moreover, algorithms can be created to optimize geometric transformations applied to a target image to maximize its similarity to a reference image. For example, many image‐registration algorithms can align frames of a video sequence in which movement artefacts are present (interframe artefacts). These algorithms can be successfully applied to align images forming a time series or z‐stack, wherein each frame is aligned with the one preceding it or with a reference frame. There are a few broad approaches to image registration. One is based on feature detection and seeks to match specific sets of landmarks (e.g. points, lines, edges) from one image to another (Ishijima *et al*., 2015; Aghayee *et al*., 2017). Aghayee and colleagues used motion correction by cell tracking. They target the brightest neurons and track its position over time using particle finding and tracking algorithm, including deformations compensation (Lorenz *et al*., 2012). Kumar and colleagues took advantage of feature detection and developed a general framework to correct for feature‐based motion using a vessel‐ness filter to identify blood vessels in the mouse pancreas (Ishijima *et al*., 2015). This method is quite robust and significantly reduces movement artefacts from multichannel time series; however, it requires considerable computation time, which reduces performance for large data sets. Using this framework, motion artefacts that occurred on a global scale (i.e. affected fields of view from one frame to another in a similar fashion) were successfully corrected with rigid transformations, whereas local deformations (i.e. present in distinct sections from each frame) were corrected with nonrigid transformations. Another approach involves nonrigid registration algorithms, which correct nonuniform localized deformations well; however, care must be taken when interpreting data, especially when imaging small motile structures, such as microglial processes or dendritic spines. Indeed, these algorithms are unlikely to correct local deformations similarly and may digitally cancel, overlook or even create them altogether. Therefore, nonrigid registration algorithms might be unsuitable for some experimental paradigms (Viergever *et al*., 2016). Alternatively, intensity‐based registration, which compares intensity patterns among images, does not require manual input to be carried out and, thus, can be easily automated (Zitová & Flusser, 2003; Rohde *et al*., 2005). Common metrics used to assess similarity include the difference of squared intensity, cross‐correlation and the sum of the log of absolute differences in addition to mutual information, which is most suitable when frames to be registered are captured with different imaging modalities (Zitová & Flusser, 2003; Dombeck *et al*., 2007; Loeckx *et al*., 2010; Jenkins *et al*., 2013; Viergever *et al*., 2016).

Given the raster‐scanning pattern used to generate images in two‐photon intravital microscopy, artefacts may occur while acquiring individual frames (intraframe artefacts). To limit the burden caused by these artefacts, different methods have been used, including the Lucas–Kanade framework, Hidden‐Markov Model and the Sequential IMaging Analysis Python package, which address artefacts on a line‐by‐line basis (Greenberg & Kerr, 2009; He *et al*., 2013; Kaifosh *et al*., 2014). Another method used to reduce intraframe artefacts is the cubature‐Kalman‐filter modelling in a nonlinear system whose constraints help the registration algorithm estimate geometrical transformations (Vercauteren *et al*., 2006; Greenberg & Kerr, 2009; Kaifosh *et al*., 2014). Moreover, Vercauteren and colleagues used the relationship between distortions and the motion that caused them to compensate for artefacts generated by a fibre‐based optical microprobe (Dunn *et al*., 2014). In addition to the custom algorithms presented above, and commercial products, front‐end software tools that are freely available have been developed to automate artefact removal and image registration in multidimensional data sets from intravital microscopy (Bethge *et al*., 2013; Soulet *et al*., 2013; Santamaría‐Pang *et al*., 2015). For comparison purposes, refer to Table 4.

**Table 4:** Algorithms and freeware available for image registration suitable for TP‐IVM

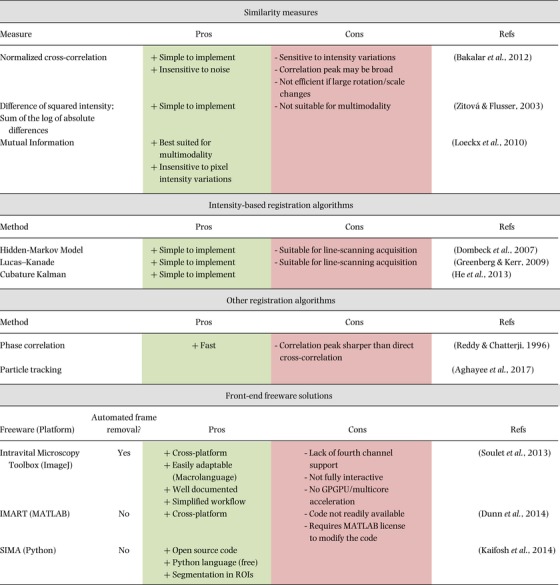

## General guidelines, tips and tricks

**Fig. 1:**
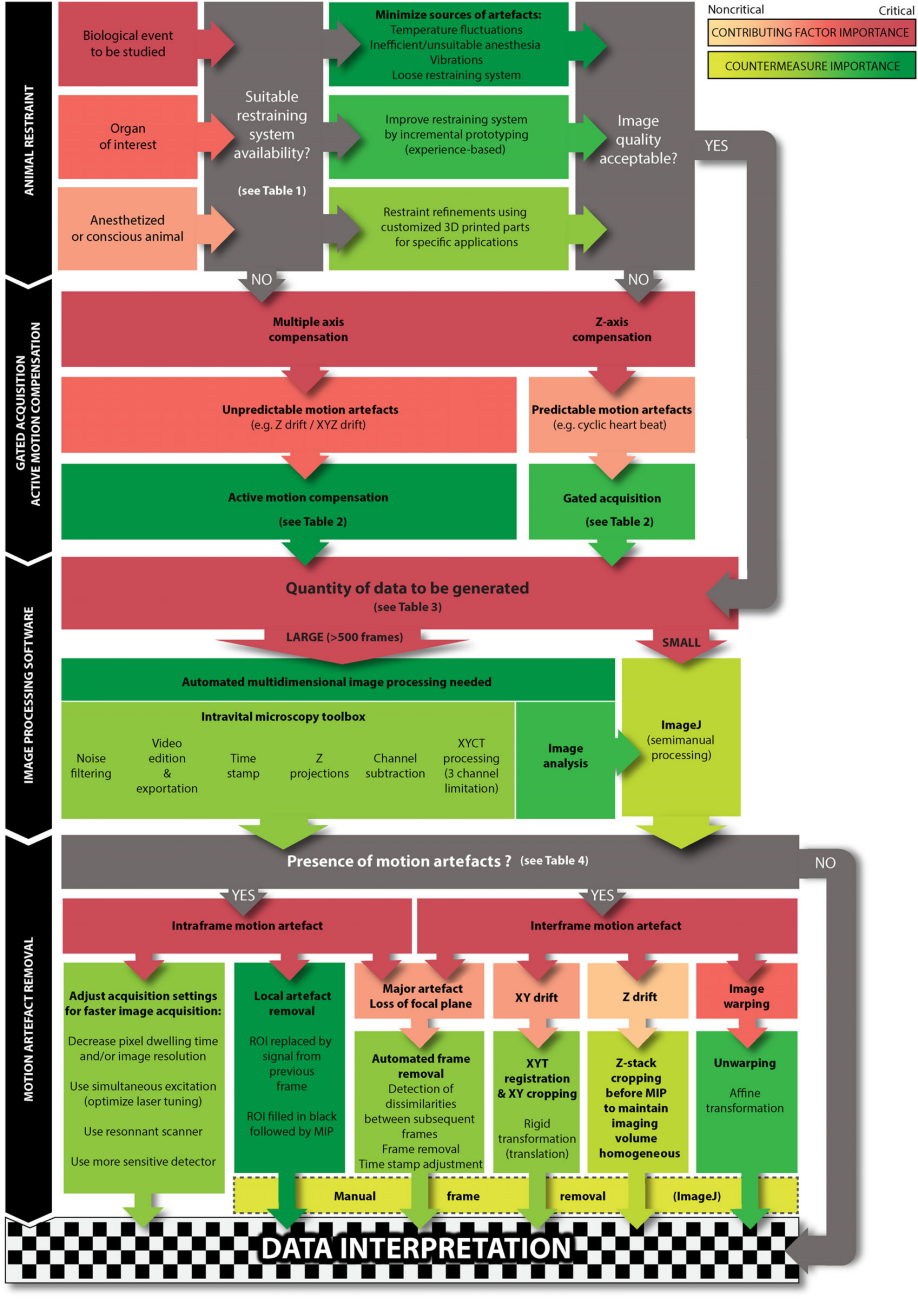
Intravital multiphoton procedures and considerations. Considering the numerous TP‐IVM applications, a single flowchart cannot be used to illustrate every possible scenario. Therefore, a general layout is depicted, emphasizing considerations related to the organ under study, typical artefacts encountered, restraining methods and possible requirements for hardware‐based active motion compensation, as well as software solutions. This figure can be used as a general guideline to reduce the impact of motion artefacts during intravital imaging sessions. MIP, maximum intensity projection; ROI, region of interest; TP‐IVM, two‐photon intravital microscopy.

Choosing a suitable two‐photon intravital microscopy approach depends on the biological event to be investigated and the organs being imaged. Here, we discuss the most feasible approaches depending on the type of organ and motion artefact, and provide examples in which various technical solutionshave been successfully used (see Fig. 1 and corresponding Tables 1, 2, 3, 4).

## Concluding remarks

Although it is virtually impossible to completely eliminate the physiological causes of movement artefacts, efficient mechanical, technical and software approaches have been developed to limit their burden and facilitate data interpretation. With the ongoing advances in molecular, optical and genetic tools that are available to access, identify and study biological mechanisms in increasingly demanding physiological environments and conditions, there is no doubt that two‐photon intravital microscopy will occupy a front‐line position in the future of biomedical research (Tomek *et al*., 2013; Pfeiffer *et al*., 2018; Soltanian‐Zadeh *et al*., 2019). Of note is that technical developments related to light access, acquisition, processing speed and signal collection efficiency will increase the amount of data being collected, thereby stressing the need for robust hardware and software solutions to efficiently manipulate the imaging data. We believe that active motion compensation in real time will become increasingly popular as computational resources and calculation power expand to enable immediate diagnosis and correction of artefacts for greater experimental throughput and more direct appreciation of generated imaging data.

## Contributions

D.S., J.L.‐P., B.A and D.D. wrote the manuscript. J.L‐P., B.A and D.S. prepared the tables and J.L‐P. and D.S. prepared the figures.

## Competing financial interests

The authors declare no competing financial interests.

## References

[jmi12880-bib-0001] Aghayee, S. , Winkowski, D.E. , Bowen, Z. *et al* (2017) Particle tracking facilitates real time capable motion correction in 2D or 3D two‐photon imaging of neuronal activity. Front. Neural Circuits 11, 56. 28860973 10.3389/fncir.2017.00056 PMC5559509

[jmi12880-bib-0002] Aguirre, A.D. , Vinegoni, C. , Sebas, M. & Weissleder, R. (2014) Intravital imaging of cardiac function at the single‐cell level. Proc. Natl. Acad. Sci. U. S. A. 111, 11257–11262. 25053815 10.1073/pnas.1401316111 PMC4128110

[jmi12880-bib-0003] Alieva, M. , Ritsma, L. , Giedt, R.J. , Weissleder, R. & van Rheenen, J. (2014) Imaging windows for long‐term intravital imaging: general overview and technical insights. Intravital 3, e29917. 28243510 10.4161/intv.29917 PMC5312719

[jmi12880-bib-0004] Andermann, M.L. , Kerlin, A.M. , Roumis, D.K. , Glickfeld, L.L. & Reid, R.C. (2011) Functional specialization of mouse higher visual cortical areas. Neuron 72, 1025–1039. 22196337 10.1016/j.neuron.2011.11.013 PMC3876958

[jmi12880-bib-0005] Aubé, B , Levesque, S.A. , Pare, A. *et al* (2014) Neutrophils mediate blood‐spinal cord barrier disruption in demyelinating neuroinflammatory diseases. J. Immunol. 193, 2438–2454. 25049355 10.4049/jimmunol.1400401

[jmi12880-bib-0006] Bakalar, M. , Schroeder, J.L. , Pursley, R. *et al* (2012) Three‐dimensional motion tracking for high‐resolution optical microscopy, in vivo. J. Microsc. 246, 237–247. 22582797 10.1111/j.1365-2818.2012.03613.x PMC3799900

[jmi12880-bib-0007] Berdyyeva, T. , Otte, S. , Aluisio, L. *et al* (2014) Zolpidem reduces hippocampal neuronal activity in freely behaving mice: a large scale calcium imaging study with miniaturized fluorescence microscope. PLoS One 9, e112068. 25372144 10.1371/journal.pone.0112068 PMC4221229

[jmi12880-bib-0008] Bethge, P. , Chéreau, R. , Avignone, E. , Marsicano, G. & Nägerl, U.V. (2013) Two‐photon excitation STED microscopy in two colors in acute brain slices. Biophys. J. 104, 778–785. 23442956 10.1016/j.bpj.2012.12.054 PMC3576543

[jmi12880-bib-0009] Birkner, A. , Tischbirek, C.H. & Konnerth, A. (2017) Improved deep two‐photon calcium imaging in vivo. Cell Calcium 64, 29–35. 28027798 10.1016/j.ceca.2016.12.005

[jmi12880-bib-0010] Botcherby, E.J. , Juškaitis, R. & Wilson, T. (2006) Scanning two photon fluorescence microscopy with extended depth of field. Opt. Commun. 268, 253–260.

[jmi12880-bib-0011] Bullen, A. (2008) Microscopic imaging techniques for drug discovery. Nat. Rev. Drug Discov. 7, 54–67. 18079755 10.1038/nrd2446

[jmi12880-bib-0012] Cao, L. , Kobayakawa, S. , Yoshiki, A. & Abe, K. (2012) High resolution intravital imaging of subcellular structures of mouse abdominal organs using a microstage device. PLoS One 7, e33876. 22479464 10.1371/journal.pone.0033876 PMC3313950

[jmi12880-bib-0013] Chen, M. , Wen, D. , Huang, S. , Gui, S. , Zhang, Z. , Lu, J. & Li, P. (2018) Laser speckle contrast imaging of blood flow in the deep brain using microendoscopy. Opt. Lett. 43, 5627–5630. 30439911 10.1364/OL.43.005627

[jmi12880-bib-0014] Chèvre, R. , González‐Granado, J.M. , Megens, R.T.A. *et al* (2014) High‐resolution imaging of intravascular atherogenic inflammation in live mice. Circ. Res. 114, 770–779. 24366169 10.1161/CIRCRESAHA.114.302590

[jmi12880-bib-0015] Coisne, C. , Lyck, R. & Engelhardt, B. (2013) Live cell imaging techniques to study T cell trafficking across the blood‐brain barrier in vitro and in vivo. Fluids Barriers CNS 10, 7. 23336847 10.1186/2045-8118-10-7 PMC3560242

[jmi12880-bib-0016] Davalos, D. & Akassoglou, K. (2012) In vivo imaging of the mouse spinal cord using two‐photon microscopy. J. Vis. Exp. e2760 10.3791/2760 22258623 PMC3369767

[jmi12880-bib-0017] Davalos, D. , Lee, J.K. , Smith, W.B. , Brinkman, B. , Ellisman, M.H. , Zheng, B. & Akassoglou, K. (2008) Stable in vivo imaging of densely populated glia, axons and blood vessels in the mouse spinal cord using two‐photon microscopy. J. Neurosci. Methods 169, 1–7. 18192022 10.1016/j.jneumeth.2007.11.011 PMC2647134

[jmi12880-bib-0018] Davalos, D. , Ryu, J.K. , Merlini, K.M. *et al* (2012) Fibrinogen‐induced perivascular microglial clustering is required for the development of axonal damage in neuroinflammation. Nat. Commun. 3, 1227. 23187627 10.1038/ncomms2230 PMC3514498

[jmi12880-bib-0019] Denk, W. , Strickler, J.H. & Webb, W.W. (1990) Two‐photon laser scanning fluorescence microscopy. Science 248, 73–76. 2321027 10.1126/science.2321027

[jmi12880-bib-0020] Diaz, L.L. , Zhang, J. & Heerdt, P.M. (2014) Comparative pharmacodynamics of pancuronium, cisatracurium, and CW002 in rabbits. J. Am. Assoc. Lab. Anim. Sci. 53, 283–289. 24827571 PMC4128567

[jmi12880-bib-0021] Dombeck, D.A. , Khabbaz, A.N. , Collman, F. , Adelman, T.L. & Tank, D.W. (2007) Imaging large‐scale neural activity with cellular resolution in awake, mobile mice. Neuron 56, 43–57. 17920014 10.1016/j.neuron.2007.08.003 PMC2268027

[jmi12880-bib-0022] Dorand, R.D. , Barkauskas, D.S. , Evans, T.A. , Petrosiute, A. & Huang, A.Y. (2014) Comparison of intravital thinned skull and cranial window approaches to study CNS immunobiology in the mouse cortex. Intravital 3, e29728. 25568834 10.4161/intv.29728 PMC4283137

[jmi12880-bib-0023] Drechsler, M. , Megens, R.T.A. , van Zandvoort, M. , Weber, C. & Soehnlein, O. (2010) Hyperlipidemia‐triggered neutrophilia promotes early atherosclerosis. Circulation 122, 1837–1845. 20956207 10.1161/CIRCULATIONAHA.110.961714

[jmi12880-bib-0024] Drew, P.J. , Shih, A.Y. , Driscoll, J.D. *et al* (2010) Chronic optical access through a polished and reinforced thinned skull. Nat. Methods 7, 981–984. 20966916 10.1038/nmeth.1530 PMC3204312

[jmi12880-bib-0025] Dunn, K.W. , Lorenz, K.S. , Salama, P. & Delp, E.J. (2014) IMART software for correction of motion artifacts in images collected in intravital microscopy. Intravital 3, e28210. 26090271 10.4161/intv.28210 PMC4469201

[jmi12880-bib-0026] Ellenbroek, S.I.J. & van Rheenen, J. (2014) Imaging hallmarks of cancer in living mice. Nat. Rev. Cancer 14, 406–418. 24854083 10.1038/nrc3742

[jmi12880-bib-0027] Evans, T.A. , Barkauskas, D.S. , Myers, J.T. & Huang, A.Y. (2014) Intravital imaging of axonal interactions with microglia and macrophages in a mouse dorsal column crush injury. J. Vis. Exp. e52228 10.3791/52228 25489963 PMC4275021

[jmi12880-bib-0028] Farrar, M.J. , Bernstein, I.M. , Schaffer, C.B. , Schlafer, D.H. , Cleland, T.A. & Fetcho, J.R. (2012) Chronic in vivo imaging in the mouse spinal cord using an implanted chamber. Nat. Methods 9, 297–302. 22266542 10.1038/nmeth.1856 PMC3429123

[jmi12880-bib-0029] Fenrich, K.K. , Weber, P. , Rougon, G. & Debarbieux, F. (2013) Long‐ and short‐term intravital imaging reveals differential spatiotemporal recruitment and function of myelomonocytic cells after spinal cord injury. J. Physiol. 591, 4895–4902. 23918770 10.1113/jphysiol.2013.256388 PMC3800461

[jmi12880-bib-0030] Figley, S.A. , Fehlings, M.G. , Burrell, K. *et al* (2013) A spinal cord window chamber model for in vivo longitudinal multimodal optical and acoustic imaging in a murine model. PLoS One 8, e58081. 23516432 10.1371/journal.pone.0058081 PMC3597636

[jmi12880-bib-0031] Fiole, D. , Deman, P. , Trescos, Y. *et al* (2014) Two‐photon intravital imaging of lungs during anthrax infection reveals long‐lasting macrophage‐dendritic cell contacts. Infect. Immun. 82, 864–872. 24478099 10.1128/IAI.01184-13 PMC3911401

[jmi12880-bib-0032] Garaschuk, O. , Milos, R.‐I. & Konnerth, A. (2006) Targeted bulk‐loading of fluorescent indicators for two‐photon brain imaging in vivo. Nat. Protoc. 1, 380–386. 17406260 10.1038/nprot.2006.58

[jmi12880-bib-0033] Ghosh, K.K. , Burns, L.D. , Cocker, E.D. *et al* (2011) Miniaturized integration of a fluorescence microscope. Nat. Methods 8, 871–878. 21909102 10.1038/nmeth.1694 PMC3810311

[jmi12880-bib-0034] Goto, K. , Kato, G. , Kawahara, I. *et al* (2013) In vivo imaging of enteric neurogenesis in the deep tissue of mouse small intestine. PLoS One 8, e54814. 23382976 10.1371/journal.pone.0054814 PMC3561410

[jmi12880-bib-0035] Greenberg, D.S. & Kerr, J.N.D. (2009) Automated correction of fast motion artifacts for two‐photon imaging of awake animals. J. Neurosci. Methods 176, 1–15. 18789968 10.1016/j.jneumeth.2008.08.020

[jmi12880-bib-0036] Grewe, B.F. & Helmchen, F. (2009) Optical probing of neuronal ensemble activity. Curr. Opin. Neurobiol. 19, 520–529. 19854041 10.1016/j.conb.2009.09.003

[jmi12880-bib-0037] Gu, L. , Kleiber, S. , Schmid, L. , Nebeling, F. , Chamoun, M. , Steffen, J. , Wagner, J. & Fuhrmann, M. (2014) Long‐term in vivo imaging of dendritic spines in the hippocampus reveals structural plasticity. J. Neurosci. 34, 13948–13953. 25319691 10.1523/JNEUROSCI.1464-14.2014 PMC6705298

[jmi12880-bib-0038] Gulati, S. , Cao, V.Y. & Otte, S. (2017) Multi‐layer cortical Ca2+ imaging in freely moving mice with prism probes and miniaturized fluorescence microscopy. J. Vis. Exp. 55579 10.3791/55579 PMC5608392 28654056

[jmi12880-bib-0039] Haft‐Javaherian, M. , Fang, L. , Muse, V. , Schaffer, C.B. , Nishimura, N.N. & Sabuncu, M.R. (2019) Deep convolutional neural networks for segmenting 3D in vivo multiphoton images of vasculature in Alzheimer disease mouse models. PLoS One 14, e0213539. 30865678 10.1371/journal.pone.0213539 PMC6415838

[jmi12880-bib-0040] Haghayegh Jahromi, N. , Tardent, H. , Enzmann, G. *et al* (2017) A novel cervical spinal cord window preparation allows for two‐photon imaging of T‐cell interactions with the cervical spinal cord microvasculature during experimental autoimmune encephalomyelitis. Front. Immunol. 8, 406. 28443093 10.3389/fimmu.2017.00406 PMC5387098

[jmi12880-bib-0041] He, T. , Xue, Z. , Alvarado, M.V. , Wong, K.K. , Xie, W. & Wong, S.T.C. (2013) Nonlinear motion compensation using cubature Kalman filter for in vivo fluorescence microendoscopy in peripheral lung cancer intervention. J. Biomed. Opt. 18, 16008. 23291716 10.1117/1.JBO.18.1.016008

[jmi12880-bib-0042] Helmchen, F. & Denk, W. (2005) Deep tissue two‐photon microscopy. Nat. Methods 2, 932–940. 16299478 10.1038/nmeth818

[jmi12880-bib-0043] Helmchen, F. , Denk, W. & Kerr, J.N.D. (2013) Miniaturization of two‐photon microscopy for imaging in freely moving animals. Cold Spring Harb. Protoc. 2013, 904–913. 24086055 10.1101/pdb.top078147

[jmi12880-bib-0044] Holtmaat, A. , Bonhoeffer, T. , Chow, D.K. *et al* (2009) Long‐term, high‐resolution imaging in the mouse neocortex through a chronic cranial window. Nat. Protoc. 4, 1128–1144. 19617885 10.1038/nprot.2009.89 PMC3072839

[jmi12880-bib-0045] Ishijima, A. , Schwarz, R.A. , Shin, S. *et al* (2015) Automated frame selection process for high‐resolution microendoscopy. J. Biomed. Opt. 20, 46014. 25919426 10.1117/1.JBO.20.4.046014 PMC4412137

[jmi12880-bib-0046] Jenkins, M.W. , Wang, Y.T. , Doughman, Y.Q. , Watanabe, M. , Cheng, Y. & Rollins, A.M. (2013) Optical pacing of the adult rabbit heart. Biomed. Opt. Express 4, 1626–1635. 24049683 10.1364/BOE.4.001626 PMC3771833

[jmi12880-bib-0047] Johannssen, H.C. & Helmchen, F. (2010) In vivo Ca2+ imaging of dorsal horn neuronal populations in mouse spinal cord. J. Physiol. 588, 3397–3402. 20660563 10.1113/jphysiol.2010.191833 PMC2988506

[jmi12880-bib-0048] Jones, J.S. , Small, D.M. & Nishimura, N. (2018) In vivo calcium imaging of cardiomyocytes in the beating mouse heart with multiphoton microscopy. Front. Physiol. 9, 969. 30108510 10.3389/fphys.2018.00969 PMC6079295

[jmi12880-bib-0049] Kaifosh, P. , Zaremba, J.D. , Danielson, N.B. & Losonczy, A. (2014) SIMA: Python software for analysis of dynamic fluorescence imaging data. Front. Neuroinformatics 8, 80. 10.3389/fninf.2014.00080 PMC4172099 25295002

[jmi12880-bib-0050] Kelly, A. , Salerno, S. , Connolly, A. *et al* (2018) Normal interventricular differences in tissue architecture underlie right ventricular susceptibility to conduction abnormalities in a mouse model of Brugada syndrome. Cardiovasc. Res. 114, 724–736. 29267949 10.1093/cvr/cvx244 PMC5915948

[jmi12880-bib-0051] Kerlin, A.M. , Andermann, M.L. , Berezovskii, V.K. & Reid, R.C. (2010) Broadly tuned response properties of diverse inhibitory neuron subtypes in mouse visual cortex. Neuron 67, 858–871. 20826316 10.1016/j.neuron.2010.08.002 PMC3327881

[jmi12880-bib-0052] Kerr, J.N.D. & Nimmerjahn, A. (2012) Functional imaging in freely moving animals. Curr. Opin. Neurobiol. 22, 45–53. 22237048 10.1016/j.conb.2011.12.002

[jmi12880-bib-0053] Kerschensteiner, M. , Schwab, M.E. , Lichtman, J.W. & Misgeld, T. (2005) In vivo imaging of axonal degeneration and regeneration in the injured spinal cord. Nat. Med. 11, 572–577. 15821747 10.1038/nm1229

[jmi12880-bib-0054] Kim, J.V. , Jiang, N. , Tadokoro, C.E. , Lafaille, J.J. , Dustin, M.L. , Liu, L. & Ransohoff, R.M. (2010) Two‐photon laser scanning microscopy imaging of intact spinal cord and cerebral cortex reveals requirement for CXCR6 and neuroinflammation in immune cell infiltration of cortical injury sites. J. Immunol. Methods 352, 89–100. 19800886 10.1016/j.jim.2009.09.007 PMC2808463

[jmi12880-bib-0055] Klinger, A. , Orzekowsky‐Schroeder, R. , Smolinski, D. *et al* (2012) Complex morphology and functional dynamics of vital murine intestinal mucosa revealed by autofluorescence 2‐photon microscopy. Histochem. Cell Biol. 137, 269–278. 22227801 10.1007/s00418-011-0905-0 PMC3278620

[jmi12880-bib-0056] Kreisel, D. , Nava, R.J. , Li, W. *et al* (2010) In vivo two‐photon imaging reveals monocyte‐dependent neutrophil extravasation during pulmonary inflammation. Proc. Natl. Acad. Sci. U. S. A. 107, 18073–18078. 20923880 10.1073/pnas.1008737107 PMC2964224

[jmi12880-bib-0057] Kumar, A.N. , Short, K.W. & Piston, D.W. (2013) A motion correction framework for time series sequences in microscopy images. Microsc. Microanal. 19, 433–450. 23410911 10.1017/S1431927612014250 PMC4135398

[jmi12880-bib-0058] Laffray, S. , Pagès, S. , Dufour, H. , De Koninck, P. , De Koninck, Y. & Côté, D. (2011) Adaptive movement compensation for in vivo imaging of fast cellular dynamics within a moving tissue. PLoS One 6, e19928. 21629702 10.1371/journal.pone.0019928 PMC3101223

[jmi12880-bib-0059] Lecoq, J. , Savall, J. , Vučinić, D. *et al* (2014) Visualizing mammalian brain area interactions by dual‐axis two‐photon calcium imaging. Nat. Neurosci. 17, 1825–1829. 25402858 10.1038/nn.3867 PMC5289313

[jmi12880-bib-0060] Lee, S. , Nakamura, Y. , Yamane, Y. , Toujo, T. , Takahashi, S. , Tanikawa, Y. & Takahashi, H. (2008a) Image stabilization for in vivo microscopy by high‐speed visual feedback control. IEEE Trans. Robot. 24, 45–54.

[jmi12880-bib-0061] Lee, S. , Ozaki, T. & Nakamura, Y. (2008b) In vivo microscope image stabilization through 3‐D motion compensation using a contact‐type sensor. In *IEEE/RSJ International Conference on Intelligent Robots and Systems*, pp. 1192–1197. 10.1109/IROS.2008.4651123

[jmi12880-bib-0062] Lee, S. , Vinegoni, C. , Feruglio, P.F. *et al* (2012) Real‐time in vivo imaging of the beating mouse heart at microscopic resolution. Nat. Commun. 3, 1054. 22968700 10.1038/ncomms2060 PMC3622400

[jmi12880-bib-0063] Lee, S. , Vinegoni, C. , Sebas, M. & Weissleder, R. (2014) Automated motion artifact removal for intravital microscopy, without a priori information. Sci. Rep. 4, 4507. 24676021 10.1038/srep04507 PMC3968488

[jmi12880-bib-0064] Li, W. , Nava, R.G. , Bribriesco, A.C. *et al* (2012) Intravital 2‐photon imaging of leukocyte trafficking in beating heart. J. Clin. Invest. 122, 2499–2508. 22706307 10.1172/JCI62970 PMC3386827

[jmi12880-bib-0065] Loeckx, D. , Slagmolen, P. , Maes, F. , Vandermeulen, D. & Suetens, P. (2010) Nonrigid image registration using conditional mutual information. IEEE Trans. Med. Imaging 29, 19–29. 19447700 10.1109/TMI.2009.2021843

[jmi12880-bib-0066] Looney, M.R. , Thornton, E.E. , Sen, D. , Lamm, W.J. , Glenny, R.W. & Krummel, M.F. (2011) Stabilized imaging of immune surveillance in the mouse lung. Nat. Methods 8, 91–96. 21151136 10.1038/nmeth.1543 PMC3076005

[jmi12880-bib-0067] Lorenz, K.S. , Salama, P. , Dunn, K.W. & Delp, E.J. (2012) Digital correction of motion artefacts in microscopy image sequences collected from living animals using rigid and nonrigid registration. J. Microsc. 245, 148–160. 22092443 10.1111/j.1365-2818.2011.03557.x PMC3856233

[jmi12880-bib-0068] Lucotte, B. & Balaban, R.S. (2014) Motion compensation for in vivo subcellular optical microscopy. J. Microsc. 254, 9–12. 24673143 10.1111/jmi.12116 PMC4229363

[jmi12880-bib-0069] Manglani, M. & McGavern, D.B. (2018) Intravital imaging of neuroimmune interactions through a thinned skull. Curr. Protoc. Immunol. 120, 24.2.1–24.2.12. 10.1002/cpim.46 PMC5844364 29512146

[jmi12880-bib-0070] Marques, P.E. , Antunes, M.M. , David, B.A. , Pereira, R.V. , Teixeira, M.M. & Menezes, G.B. (2015) Imaging liver biology in vivo using conventional confocal microscopy. Nat. Protoc. 10, 258–268. 25569332 10.1038/nprot.2015.006

[jmi12880-bib-0071] Matsuura, R. , Miyagawa, S. , Miyagawa, S. , *et al* (2018) Intravital imaging with two‐photon microscopy reveals cellular dynamics in the ischeamia‐reperfused rat heart. Sci. Rep. 8, 15991. 30375442 10.1038/s41598-018-34295-w PMC6207786

[jmi12880-bib-0072] Megens, R.T.A. , Kemmerich, K. , Pyta, J. , Weber, C. & Soehnlein, O. (2011) Intravital imaging of phagocyte recruitment. Thromb. Haemost. 105, 802–810. 21437362 10.1160/TH10-11-0735

[jmi12880-bib-0073] Megens, R.T.A. , Reitsma, S. , Prinzen, L. *et al* (2010) In vivo high‐resolution structural imaging of large arteries in small rodents using two‐photon laser scanning microscopy. J. Biomed. Opt. 15, 011108. 20210434 10.1117/1.3281672

[jmi12880-bib-0074] Mizrahi, A. , Crowley, J. C. , Shtoyerman, E. & Katz, L.C. (2004) High‐resolution in vivo imaging of hippocampal dendrites and spines. J. Neurosci. 24, 3147–3151. 15056694 10.1523/JNEUROSCI.5218-03.2004 PMC6730023

[jmi12880-bib-0075] Mizuno, R. , Kamioka, Y. , Kabashima, K. *et al* (2014) In vivo imaging reveals PKA regulation of ERK activity during neutrophil recruitment to inflamed intestines. J. Exp. Med. 211, 1123–1136. 24842369 10.1084/jem.20132112 PMC4042632

[jmi12880-bib-0076] Motegi, Y . *et al* (2020) Confocal and multiphoton calcium imaging of the enteric nervous system in anesthetized mice. Neurosci. Res. 151, 53–60. 10.1016/j.neures 30790590

[jmi12880-bib-0077] Nadella, K.M.N.S. , Roš, H. , Baragli, C. *et al* (2016) Random‐access scanning microscopy for 3D imaging in awake behaving animals. Nat. Methods 13, 1001–1004. 27749836 10.1038/nmeth.4033 PMC5769813

[jmi12880-bib-0078] Nelson, N.A. , Wang, X. , Cook, D. , Carey, E.M. & Nimmerjahn, A. (2019) Imaging spinal cord activity in behaving animals. Exp. Neurol. 320, 112974. 31175843 10.1016/j.expneurol.2019.112974 PMC7840222

[jmi12880-bib-0079] Niesner, R.A. & Hauser, A.E. (2011) Recent advances in dynamic intravital multi‐photon microscopy. Cytom. J. Int. Soc. Anal. Cytol. 79, 789–798. 10.1002/cyto.a.21140 21905212

[jmi12880-bib-0080] Nikić, I. , Merkler, D. , Sorbara, C. *et al* (2011) A reversible form of axon damage in experimental autoimmune encephalomyelitis and multiple sclerosis. Nat. Med. 17, 495–499. 21441916 10.1038/nm.2324

[jmi12880-bib-0081] Odoardi, F. , Kawakami, N. , Klinkert, W.E.F. , Wekerle, H. & Flügel, A. (2007) Blood‐borne soluble protein antigen intensifies T cell activation in autoimmune CNS lesions and exacerbates clinical disease. Proc. Natl. Acad. Sci. U. S. A. 104, 18625–18630. 18000062 10.1073/pnas.0705033104 PMC2141827

[jmi12880-bib-0082] Packer, A.M. , Russell, L. E. , Dalgleish, H.W.P. & Häusser, M. (2015) Simultaneous all‐optical manipulation and recording of neural circuit activity with cellular resolution in vivo. Nat. Methods 12, 140–146. 25532138 10.1038/nmeth.3217 PMC4933203

[jmi12880-bib-0083] Park, H. , You, N. , Lee, J. & Suh, M. (2019) Longitudinal study of hemodynamics and dendritic membrane potential changes in the mouse cortex following a soft cranial window installation. Neurophotonics 6, 015006. 30820438 10.1117/1.NPh.6.1.015006 PMC6387987

[jmi12880-bib-0084] Paukert, M. & Bergles, D.E. (2012) Reduction of motion artifacts during in vivo two‐photon imaging of brain through heartbeat triggered scanning. J. Physiol. 590, 2955–2963. 22508962 10.1113/jphysiol.2012.228114 PMC3406383

[jmi12880-bib-0085] Pfeiffer, T. , Poll, S. , Bancelin, S. *et al* (2018) Chronic 2P‐STED imaging reveals high turnover of dendritic spines in the hippocampus in vivo. eLife 7, e34700. 29932052 10.7554/eLife.34700 PMC6014725

[jmi12880-bib-0086] Pittet, M.J. & Weissleder, R. (2011) Intravital imaging. Cell 147, 983–991. 22118457 10.1016/j.cell.2011.11.004 PMC3824153

[jmi12880-bib-0087] Reddy, B.S. & Chatterji, B.N. (1996) An FFT‐based technique for translation, rotation, and scale‐invariant image registration. IEEE Trans. Image Process. 5, 1266–1271. 18285214 10.1109/83.506761

[jmi12880-bib-0088] Ritsma, L. , Ellenbroek, S.I.J. , Zomer, A. *et al* (2014) Intestinal crypt homeostasis revealed at single‐stem‐cell level by in vivo live imaging. Nature 507, 362–365. 24531760 10.1038/nature12972 PMC3964820

[jmi12880-bib-0089] Rohde, G.K. , Dawant, B.M. & Lin, S.‐F. (2005) Correction of motion artifact in cardiac optical mapping using image registration. IEEE Trans. Biomed. Eng. 52, 338–341. 15709673 10.1109/TBME.2004.840464

[jmi12880-bib-0090] Sanderson, M.J. , Smith, I. , Parker, I. & Bootman, M.D. (2014) Fluorescence microscopy. Cold Spring Harb. Protoc. 2014, pdb.top071795–pdb.top071795. 25275114 10.1101/pdb.top071795 PMC4711767

[jmi12880-bib-0091] Santamaría‐Pang, A. , Hernandez‐Herrera, P. , Papadakis, M. , Saggau, P. & Kakadiaris, I.A. (2015) Automatic morphological reconstruction of neurons from multiphoton and confocal microscopy images using 3D tubular models. Neuroinformatics 13, 297–320. 25631538 10.1007/s12021-014-9253-2

[jmi12880-bib-0092] Sato, M. , Motegi, Y. , Yagi, S. *et al* (2017) Fast varifocal two‐photon microendoscope for imaging neuronal activity in the deep brain. Biomed. Opt. Express 8, 4049–4060. 28966846 10.1364/BOE.8.004049 PMC5611922

[jmi12880-bib-0093] Sato, T.R. , Gray, N.W. , Mainen, Z.F. & Svoboda, K. (2007) The functional microarchitecture of the mouse barrel cortex. PLoS Biol. 5, e189. 17622195 10.1371/journal.pbio.0050189 PMC1914403

[jmi12880-bib-0094] Sawinski, J. , Wallace, D.J. , Greenberg, D.S. *et al* (2009) Visually evoked activity in cortical cells imaged in freely moving animals. Proc. Natl. Acad. Sci. U. S. A. 106, 19557–19562. 19889973 10.1073/pnas.0903680106 PMC2773198

[jmi12880-bib-0095] Schaffran, B. , Hilton, B.J. & Bradke, F. (2019) Imaging in vivo dynamics of sensory axon responses to CNS injury. Exp. Neurol. 317, 110–118. 10.1016/j.expneurol.2019.02.010 30794766

[jmi12880-bib-0096] Schroeder, J.L. , Luger‐Hamer, M. , Pursley, R. *et al* (2010) Short communication: subcellular motion compensation for minimally invasive microscopy, in vivo: evidence for oxygen gradients in resting muscle. Circ. Res. 106, 1129–1133. 20167928 10.1161/CIRCRESAHA.109.211946 PMC3209509

[jmi12880-bib-0097] Scott, G. , Fagerholm, E.D. , Mutoh, H. *et al* (2014) Voltage imaging of waking mouse cortex reveals emergence of critical neuronal dynamics. J. Neurosci. 34, 16611–16620. 25505314 10.1523/JNEUROSCI.3474-14.2014 PMC4261090

[jmi12880-bib-0098] Sekiguchi, K.J . *et al* (2016) Imaging large‐scale cellular activity in spinal cord of freely behaving mice. Nat. Commun. 7, 11450. 27121084 10.1038/ncomms11450 PMC4853475

[jmi12880-bib-0099] Shih, A.Y. , Mateo, C. , Drew, P.J. , Tsai, P.S. & Kleinfeld, D. (2012) A polished and reinforced thinned‐skull window for long‐term imaging of the mouse brain. J. Vis. Exp. 3742 10.3791/3742 22433225 PMC3460568

[jmi12880-bib-0100] Soltanian‐Zadeh, S. , Sahingur, K. , Blau, S. , Gong, Y. & Farsiu, S. (2019) Fast and robust active neuron segmentation in two‐photon calcium imaging using spatiotemporal deep learning. Proc. Natl. Acad. Sci. U. S. A. 116, 8554–8563. 10.1073/pnas.1812995116 30975747 PMC6486774

[jmi12880-bib-0101] Song, A. , Charles, A.S. , Koay, S.A. *et al* (2017) Volumetric two‐photon imaging of neurons using stereoscopy (vTwINS). Nat. Methods 14, 420. 28319111 10.1038/nmeth.4226 PMC5551981

[jmi12880-bib-0102] Soulet, D. , Paré, A. , Coste, J. & Lacroix, S. (2013) Automated filtering of intrinsic movement artifacts during two‐photon intravital microscopy. PLoS ONE 8, e53942. 23326545 10.1371/journal.pone.0053942 PMC3543396

[jmi12880-bib-0103] St‐Pierre, F. , Marshall, J.D. , Yang, Y. *et al* (2014) High‐fidelity optical reporting of neuronal electrical activity with an ultrafast fluorescent voltage sensor. Nat. Neurosci. 17, 884–889. 24755780 10.1038/nn.3709 PMC4494739

[jmi12880-bib-0104] Tomek, J. , Novak, O. & Syka, J. (2013) Two‐photon processor and SeNeCA: a freely available software package to process data from two‐photon calcium imaging at speeds down to several milliseconds per frame. J. Neurophysiol. 110, 243–256. 23576700 10.1152/jn.00087.2013

[jmi12880-bib-0105] Ueki, H. , Wang, I.H. , Fukuyama, S. *et al* (2018) In vivo imaging of the pathophysiological changes and neutrophil dynamics in influenza virus‐infected mouse lungs. Proc. Natl. Acad. Sci. U. S. A. 115, E6622–E6629. 29941581 10.1073/pnas.1806265115 PMC6048509

[jmi12880-bib-0106] Vercauteren, T. , Perchant, A. , Malandain, G. , Pennec, X. & Ayache, N. (2006) Robust mosaicing with correction of motion distortions and tissue deformations for in vivo fibered microscopy. Med. Image Anal. 10, 673–692. 16887375 10.1016/j.media.2006.06.006

[jmi12880-bib-0107] Veres, T.Z. , Kopcsányi, T. , Tirri, M. *et al* (2017b) Intubation‐free in vivo imaging of the tracheal mucosa using two‐photon microscopy. Sci. Rep. 7, 694. 28386104 10.1038/s41598-017-00769-6 PMC5429620

[jmi12880-bib-0108] Veres, T.Z. , Kopcsányi, T. , Van Panhuys, N. *et al* (2017a) Allergen‐induced CD4 ^+^ T cell cytokine production within airway mucosal dendritic cell–T cell clusters drives the local recruitment of myeloid effector cells. J. Immunol. 198, 895–907. 27903737 10.4049/jimmunol.1601448 PMC5225021

[jmi12880-bib-0109] Viergever, M.A. , Maintz, J.B.A. , Klein, S. , Murphy, K. , Staring, M. & Pluim, J.P.W. (2016) A survey of medical image registration ‐ under review. Med. Image Anal. 33, 140–144. 27427472 10.1016/j.media.2016.06.030

[jmi12880-bib-0110] Vinegoni, C. , Lee, S. , Feruglio, P.F. & Weissleder, R. (2014) Advanced motion compensation methods for intravital optical microscopy. IEEE J. Sel. Top. Quantum Electron. Publ. IEEE Lasers Electro‐Opt. Soc. 20, 83–91. 10.1109/JSTQE.2013.2279314 PMC3832946 24273405

[jmi12880-bib-0111] Xu, C. , Shen, Y. , Littman, D.R. , Dustin, M.L. & Velázquez, P. (2012) Visualization of mucosal homeostasis via single‐ and multiphoton intravital fluorescence microscopy. J. Leukoc. Biol. 92, 413–419. 22457365 10.1189/jlb.0711344 PMC3427606

[jmi12880-bib-0112] Xu, H.‐T. , Pan, F. , Yang, G. & Gan, W.‐B. (2007) Choice of cranial window type for in vivo imaging affects dendritic spine turnover in the cortex. Nat. Neurosci. 10, 549–551. 17417634 10.1038/nn1883

[jmi12880-bib-0113] Yildirim, M. , Sugihara, H. , So, P.T. C. & Sur, M. (2019) Functional imaging of visual cortical layers and subplate in awake mice with optimized three‐photon microscopy. Nat. Commun. 10, 177. 30635577 10.1038/s41467-018-08179-6 PMC6329792

[jmi12880-bib-0114] Zhang, L. , Lapierre, A. , Roy, B. *et al* (2012) Imaging glioma initiation in vivo through a polished and reinforced thin‐skull cranial window. J. Vis. Exp. e4201 10.3791/4201 PMC3529512 23207870

[jmi12880-bib-0115] Zitová, B. & Flusser, J. (2003) Image registration methods: a survey. Image Vis. Comput. 21, 977–1000.

[jmi12880-bib-0116] Ziv, Y. , Burns, L.D. , Cocker, E.D. *et al* (2013) Long‐term dynamics of CA1 hippocampal place codes. Nat. Neurosci. 16, 264–266. 23396101 10.1038/nn.3329 PMC3784308

[jmi12880-bib-0117] Zong, W. , Wu, R. , Li, M. *et al* (2017) Fast high‐resolution miniature two‐photon microscopy for brain imaging in freely behaving mice. Nat. Methods 14, 713–719. 28553965 10.1038/nmeth.4305

